# Possible Contrast Media Reduction with Low keV Monoenergetic Images in the Detection of Focal Liver Lesions: A Dual-Energy CT Animal Study

**DOI:** 10.1371/journal.pone.0133170

**Published:** 2015-07-23

**Authors:** Yong Eun Chung, Je Sung You, Hye-Jeong Lee, Joon Seok Lim, Hye Sun Lee, Song-Ee Baek, Myeong-Jin Kim

**Affiliations:** 1 Department of Radiology, Severance Hospital, Research Institute of Radiological Science, Yonsei University College of Medicine, Seoul, Korea; 2 Department of Emergency medicine, Severance Hospital, Yonsei University College of Medicine, Seoul, Korea; 3 Biostatistics Collaboration Unit, Department of Research Affairs, Yonsei University College of Medicine, Seoul, Korea; Katholieke Universiteit Leuven, BELGIUM

## Abstract

**Objective:**

To investigate the feasibility of dual-energy CT for contrast media (CM) reduction in the diagnosis of hypervascular and hypovascular focal liver lesions (FLL).

**Subjects and Methods:**

The Institutional Animal Care and Use Committee approved this study. VX2 tumors were implanted in two different segments of the liver in 13 rabbits. After 2 weeks, two phase contrast enhanced CT scans including the arterial phase (AP) and portal-venous phase (PVP) were performed three times with 24-hour intervals with three different concentrations of iodine, 300 (I_300_), 150 (I_150_) and 75 mg I/mL (I_75_). The mean HU and standard deviation (SD) were measured in the liver, the hypervascular portion of the VX2 tumor which represented hypervascular tumors, and the central necrotic area of the VX2 tumor which represented hypovascular tumors in 140kVp images with I_300_ as a reference standard and in monoenergetic images (between 40keV and 140keV) with I_150_ and I_75_. The contrast-to-noise ratio (CNR) for FLLs and the ratio of the CNRs (CNR_ratio_) between monoenergetic image sets with I_150_ and I_75_, and the reference standard were calculated.

**Results:**

For hypervascular lesions, the CNR_ratio_ was not statistically different from 1.0 between 40keV and 70keV images with I_150_, whereas the CNR_ratio_ was significantly lower than 1.0 in all keV images with I_75_. For hypovascular lesions, the CNR_ratio_ was similar to or higher than 1.0 between 40keV and 80keV with I_150_ and between 40keV and 70keV with I_75_.

**Conclusions:**

With dual-energy CT, the total amount of CM might be halved in the diagnosis of hypervascular FLLs and reduced to one-fourth in the diagnosis of hypovascular FLLs, while still preserving CNRs.

## Introduction

Similar to statistics compiled during the early eighties, contrast-induced nephropathy (CIN) is still found to be the third leading cause of hospital-acquired acute renal insufficiency and it accounts for 11–12% of all hospital-acquired acute renal insufficiency cases [1–3]. CIN is reported to have an in-hospital mortality rate of 6–14% in spite of unremitting efforts to prevent its development [1, 2]. It is also a predisposing factor for both future kidney function loss and long-term adverse events such as death, stroke, myocardial infarction, and other cardiac and kidney diseases [4, 5]. While the pathophysiology of CIN is not yet completely understood, direct cytotoxicity to endothelial and tubular cells, high viscosity, and high osmolarity of contrast media (CM) are thought to play major roles in the development of CIN [3]. Although there has been extensive study on ways to prevent CIN, the only methods proven to be effective are extracellular volume expansion and the use of low- or iso-osmolar iodinated CM rather than high-osmolar iodinated CM [6–8].

In terms of CM doses, the administered CM dose has been positively correlated with CIN risk and a higher dose of CM was reported to even increase in-hospital mortality [9, 10]. Hence, there have been attempts to reduce CM dose during CT angiography by applying the low tube voltage–high tube current technique [11–13]. As tube voltage decreases, it becomes closer to the k-edge of iodine and the photoelectric absorption of iodine increases, resulting in increased Hounsfield Units (HU) of iodine on CT [14–16]. Hence, with the low tube voltage–high tube current technique, iodine manifests with higher HU values than it does with the standard CT technique, even with the same CM dose. However, it is more difficult to apply this technique to the liver because the liver is enhanced less than the aorta or artery after contrast enhancement, resulting in a lower contrast-to-noise ratio (CNR) in liver CT compared to CT angiography. Therefore, previous studies regarding focal liver lesion (FLL) evaluation with the low tube voltage–high tube current technique focused on improving the CNR or lesion conspicuity, rather than on decreasing CM dose [17–20].

Gemstone spectral imaging (GSI) dual-energy CT can almost simultaneously acquire image data from two different tube voltages via rapid kVp switching (80 kVp and 140 kVp; switching delay, 0.25 msec) and can provide monoenergetic image sets between 40 keV and 140 keV through mathematical computation [21]. In lower keV images, a lower concentration of iodine can have similar HUs to higher concentrations of iodine in standard kVp images [13, 15]. Based on these reports, we assumed that the CNR of focal hypervascular and hypovascular hepatic lesions might be non-inferior in lower keV images even with CM doses of lower iodine concentration which suggest a smaller total amount of iodinated CM if the injection duration is fixed, compared to standard kVp images with CM of standard iodine concentrations. The purpose of this study was to investigate the feasibility of GSI dual-energy CT (DECT) for iodinated CM reduction in the diagnosis of hypervascular and hypovascular focal liver lesions.

## Materials and Method

The Institutional Animal Care and Use Committee of Yonsei University College of Medicine reviewed and approved all of this study’s protocols according to the “Guide for the Care and Use of Laboratory Animals.”

### Preparation of the animal model

The VX2 carcinoma model was adapted for this animal study. The blood supply of VX2 carcinoma is similar to that of hepatocellular carcinoma in humans, and it shows a hypervascular nature on contrast enhanced CT [22]. Furthermore, VX2 carcinoma grows rapidly and central necrosis commonly develops as it increases in size [23]. Hence, the model can represent both hypervascular and hypovascular tumors in the liver. Thirteen male New Zealand white rabbits (weight: 3.0–3.9 kg, DooYeal Biotech, Seoul, Korea) were housed in a metal cage with access to food and water ad libitum during the experiment. The rabbits were anesthetized with a mixture of tiletamine-zolazepam (15 mg/kg; Zoletil; Virbac Laboratories, Carros, France) and xylazine (5 mg/kg; Rompun; Bayer Korea, Seoul, Korea) given as an intramuscular injection at the thigh. After anesthesia, abdomen hair was shaved. An approximately 2 cm-sized midline incision was made 2 cm below the xyphoid process. After careful exposure of the liver, a 1 mm^3^ chip of VX2 carcinoma was implanted in the subcapsular area of the liver using a very fine point curved (bent at 90 degrees) forcep. In each rabbit, two VX2 carcinoma chips were implanted in different segments of the liver. After tumor implantation, the abdominal wall was closed with layer by layer sutures. To prevent post-operative infection, enrofloxacin (5 mg/kg; Bayrtil, Bayer Korea, Seoul, Korea) was injected subcutaneously twice a day for 5 days. For pain management, ketorolac tromethamine (0.5 mg/kg; Keromin, Hana Pham, Seoul, Korea) was injected intravenously once before surgery and once after surgery. All rabbits were housed again and raised 14–18 days to allow tumor growth.

### Determination of optimal scan delay

Hypervascular FLL is usually evaluated on the arterial dominant phase (AP) and hypovascular FLL is evaluated on portal venous phase (PVP) because the CNR is highest on these phases, respectively. The optimal scan delay after administration of contrast media for both the AP and PVP have been extensively studied for humans and 35–45 sec for the AP and 65–70 sec for the PVP is widely accepted as an optimal scan delay [24]. However, the hemodynamic status of rabbits might be different from humans. Therefore, the optimal scan delay must be first determined for both the AP and PVP in rabbits. All CT scans were performed with a GSI dual-energy 64-detector CT scanner (Discovery CT750 HD; GE Healthcare, Milwaukee, WI, USA). A single-location cine CT scanning was performed and the level of perfusion CT was adjusted to include the liver, portal vein and abdominal aorta. The perfusion CT protocol was summarized in Table 1. CM was injected by power injector via the ear vein in the amount of 2 mL/kg for 7 seconds, followed by a 7 mL saline flush with an injection rate of 1 mL/sec. The perfusion scan was initiated 5 seconds after CM administration (iohexol, Omnipaque 300, GE Healthcare, Cork, Ireland) and continued thereafter for 60 seconds. After completion of the perfusion scan, a region of interest (ROI) was drawn within the aorta, portal vein, the right lobe of the liver parenchyma, and paraspinal muscle. The HU value was measured three times in different slices.

**Table 1:** CT parameters.

	Perfusion CT	Dual energy CT
GSI mode	off	on
Scan mode	Cine mode	helical
Rotation time (sec)	1	0.6
Detector coverage (mm)	40	40
Slice thickness (mm)	2.5	2.5
Cinetime between images (sec)	1	-
Scan field of view	small body	medium body
Tube voltage	120 kVp	fast kV-switching between 80 and 140 kVp
Tube current	150 mA	less than 630 mA
Pitch	-	0.984:1

### CT protocol

About 2 weeks after tumor implantation, two phase contrast enhanced CT scans including the AP and PVP were performed with pre-determined scan delays. Three consecutive CT scans with different iodine concentrations of CM were performed with 24-hour intervals in each tumor-implanted rabbit. First, a CM (iohexol, Omnipaque 300, GE Healthcare, Cork, Ireland) with a concentration of 300 mg I/mL was administrated as the reference standard. After that, the contrast media was diluted with normal saline and its concentration was adjusted to 150 mg I/mL (I_150_) and 75 mg I/mL (I_75_). The CM was injected via the ear vein using a power injector in the amount of 2 ml/kg, with a fixed injection duration of 7 seconds, followed by a 7 ml saline flush. The CT parameters were summarized in Table 1.

### Image analysis

Acquired CT data were transferred to a workstation (GE Avantage; GE Healthcare) and the mean HU and standard deviation (SD) was measured in the liver, the hypervascular portion of the VX2 tumor in the AP which represented hypervascular tumors, and the central necrotic area of the VX2 tumor in the PVP which represented hypovascular tumors in 140 kVp images with I_300_ as the reference standard. Afterwards, monoenergetic (keV) images were made for energy levels between 40 and 140 keV with intervals of 10 keV, using dedicated software (Gemstone Spectral Imaging (GSI) Viewer, GE Healthcare) for I_150_ and I_75_ image data. The mean HU and SD were measured in the monoenergetic images at the same location as the reference standard image sets. The location of the ROI was carefully selected and drawn to be as large as possible without including adjacent vessels or other structures. All measurements were performed three times and average values were calculated. First, the tumor to liver contrast (TLC) was calculated with the following equation:

Hypervascular lesion (TLC_H_): HU of the hypervascular area of the VX2 tumor in the AP–HU of the liver in the AP

Hypovascular lesion: (TLC_L_): HU of the liver in the PVP—HU of the central hypovascular area of the VX2 tumor in the PVP

After the TLC was calculated, the CNR was calculated with the following equation:

$${\text{CNR}\;}_{\text{H}}=\frac{{\text{TLC}\;}_{\text{H}}}{\text{SD of the liver in the AP}}{\;\text{CNR}\;}_{\text{L}}=\frac{{\text{TLC}\;}_{\text{L}}}{\text{SD of the liver in the PVP}}$$

All calculations were made both in 140 kVp image sets with I_300_ for the reference standard, and in monoenergetic image sets (40 keV-140 keV, with intervals of 10 keV) with I_150_ and I_75_. Finally, the ratio of CNR (CNR_ratio_) was calculated for both hypervascular (CNR_Hratio_) and hypovascular (CNR_Lratio_) lesions with the following equation:

$${\text{CNR}}_{\text{ratio}}=\frac{{\text{CNR}}_{\text{I}75\;\text{or I}150\;\text{in monochromatic image sets bewteen}\;40–140\text{keV}}}{{\text{CNR}}_{\text{I}300\;\text{in}\;140\;\text{kVp}}}$$

A CNR_ratio_ that was significantly larger than 1.0 or no different from 1.0 was thought to suggest a CNR in monoenergetic images that was non-inferior to that of reference standard images.

### Statistical analysis

The one-sample t-test was used to compare the CNR_ratio_ and 1. After this step, the Bonferroni correction was performed by multiplying 11 to the calculated P-value to prevent an increase in type I errors during multiple comparisons between the 11 monochromatic image sets and 1 for each concentration of contrast media. All statistical analyses were performed by a biostatistician (H.S.L) using SAS version 9.2 (SAS institute Inc., Cary, NC, USA). A *P* value of less than 0.05 was considered statistically significant.

## Results

### Determination of optimal scan delay

**Fig 1:**
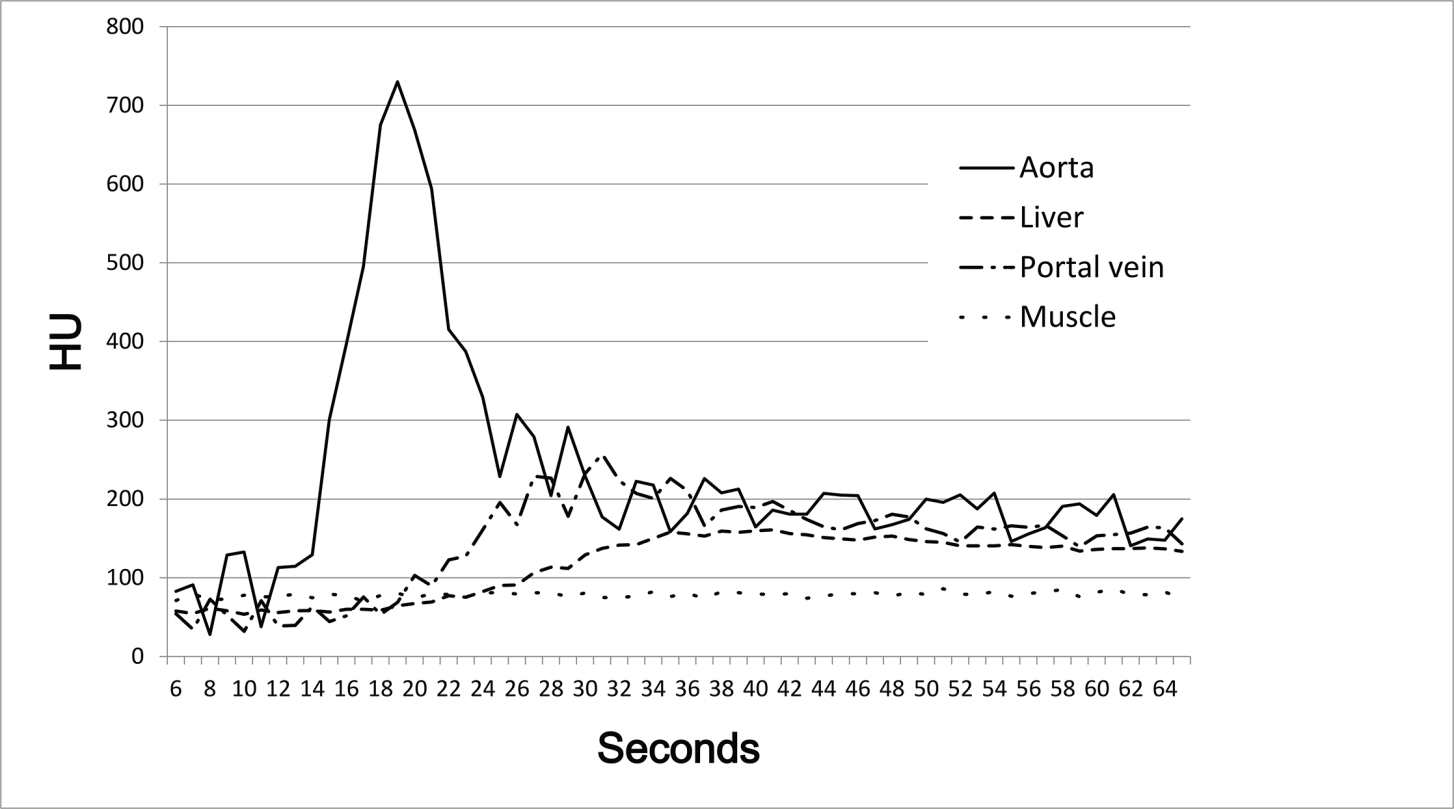
Aorta and liver showed peak enhancement at 19 seconds and 38–41 seconds after the initiation of the contrast media administration, respectively.

The average peak enhancement of the aorta and liver was observed 19 seconds and 38–41 seconds after CM administration was initiated, respectively (Fig 1). Because the peak enhancement of a hypervascular tumor is followed by that of the aorta [25], the scan delay of the AP was determined as 23 seconds. The PVP was obtained 14 seconds after the AP ended.

### Image analysis

Among the 13 rabbits, 23 of 26 implanted VX2 tumors (mean ± SD, 13.9 mm ± 4.3 mm; range, 7.8 mm—26.6 mm) grew successfully. In three rabbits, only one of the two implanted VX2 tumors grew.

**Fig 2:**
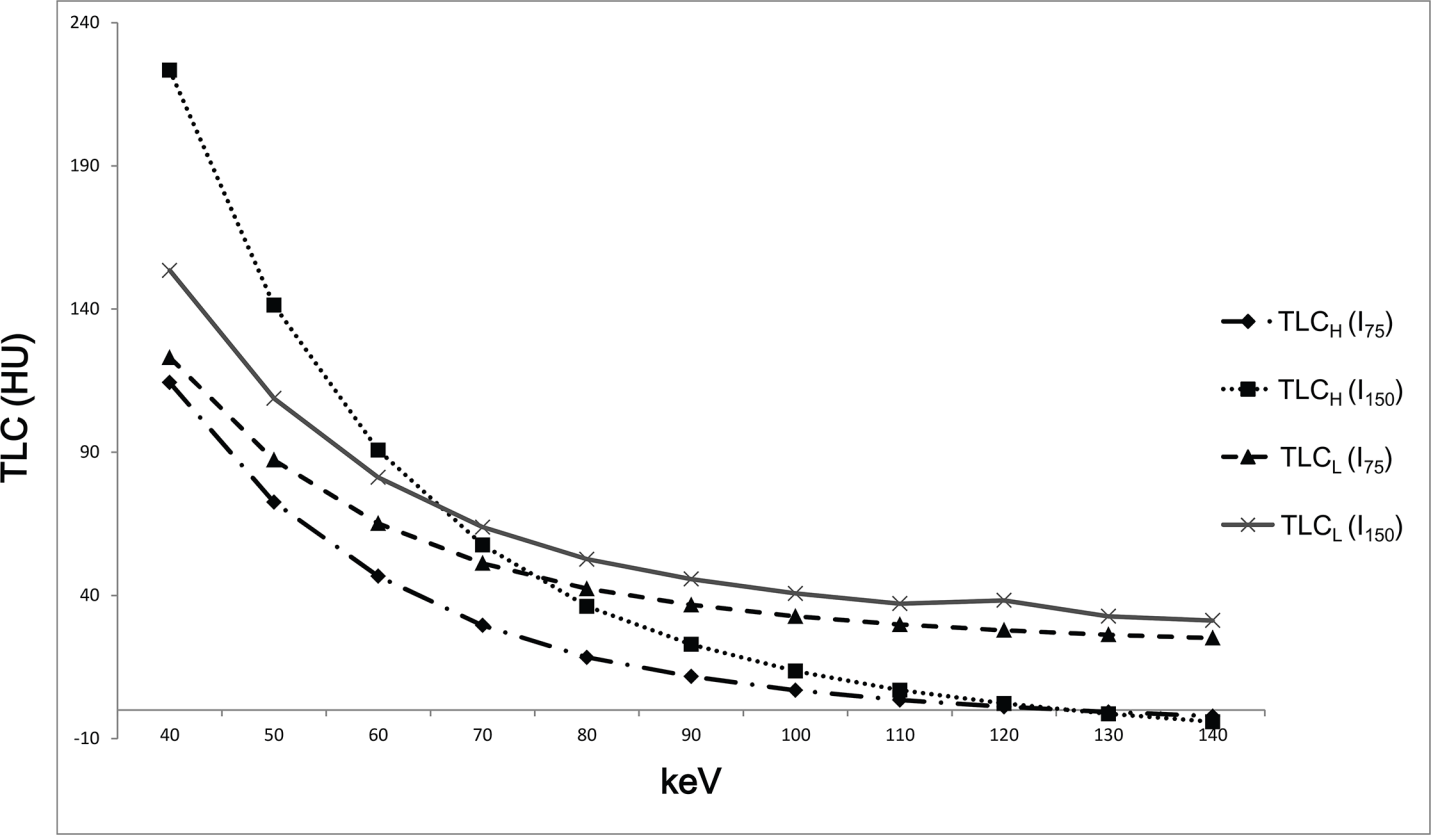
Changes of TLC according to keV. Both TLC_H_ and TLC_L_ increased as keV decreased for all iodine concentrations of CM.

**Fig 3:**
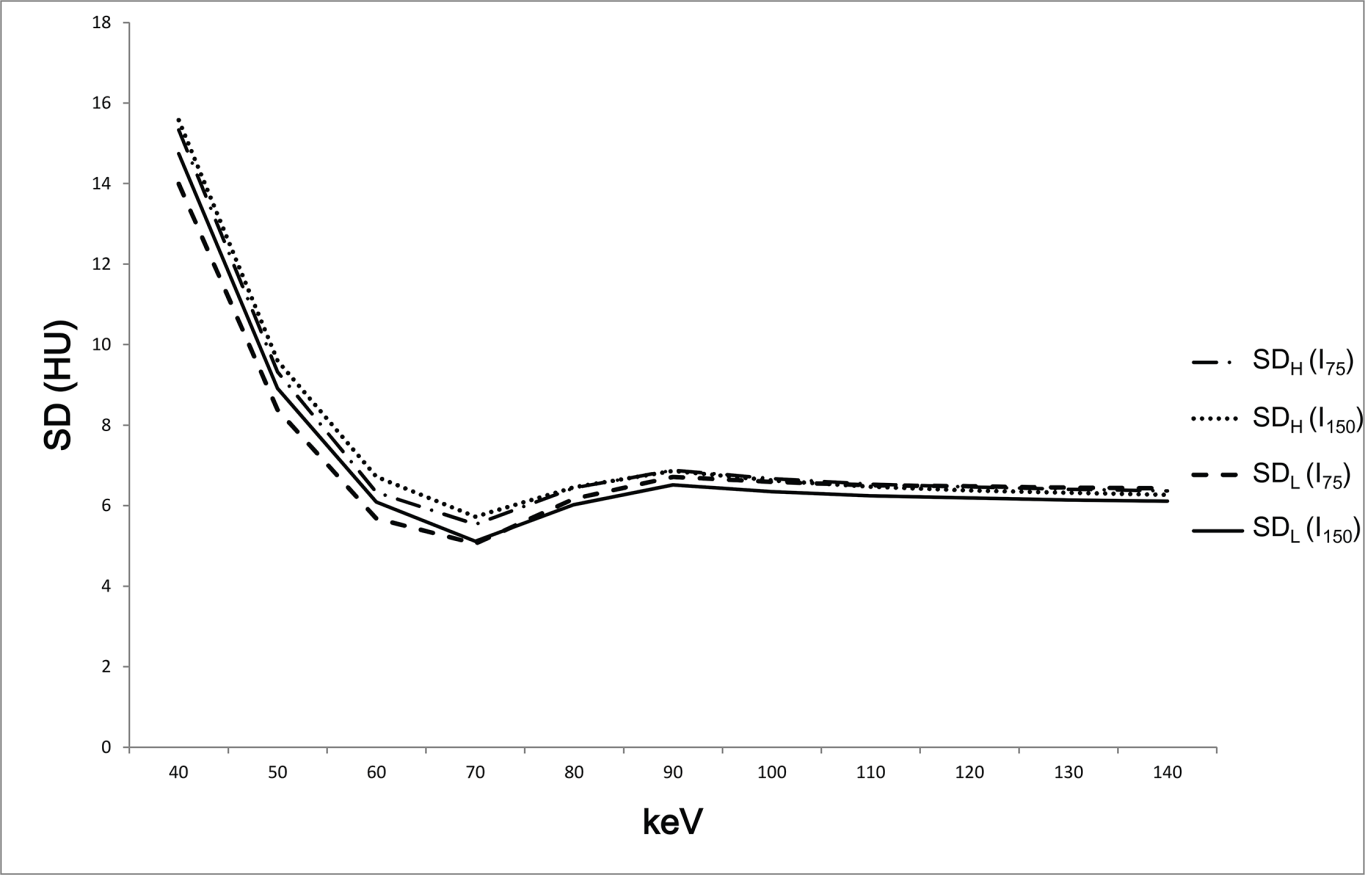
The SD showed its lowest value in 70 keV in both the AP and HVP for all concentrations of CM.

Both TLC_H_ and TLC_L_ increased as keV decreased with both I_75_ and I_150_ (Fig 2). The SD was lowest in 70 keV and rapidly increased in keV values lower than 70 keV in both the AP and PVP and with both I_75_ and I_150_ (Fig 3).

**Fig 4:**
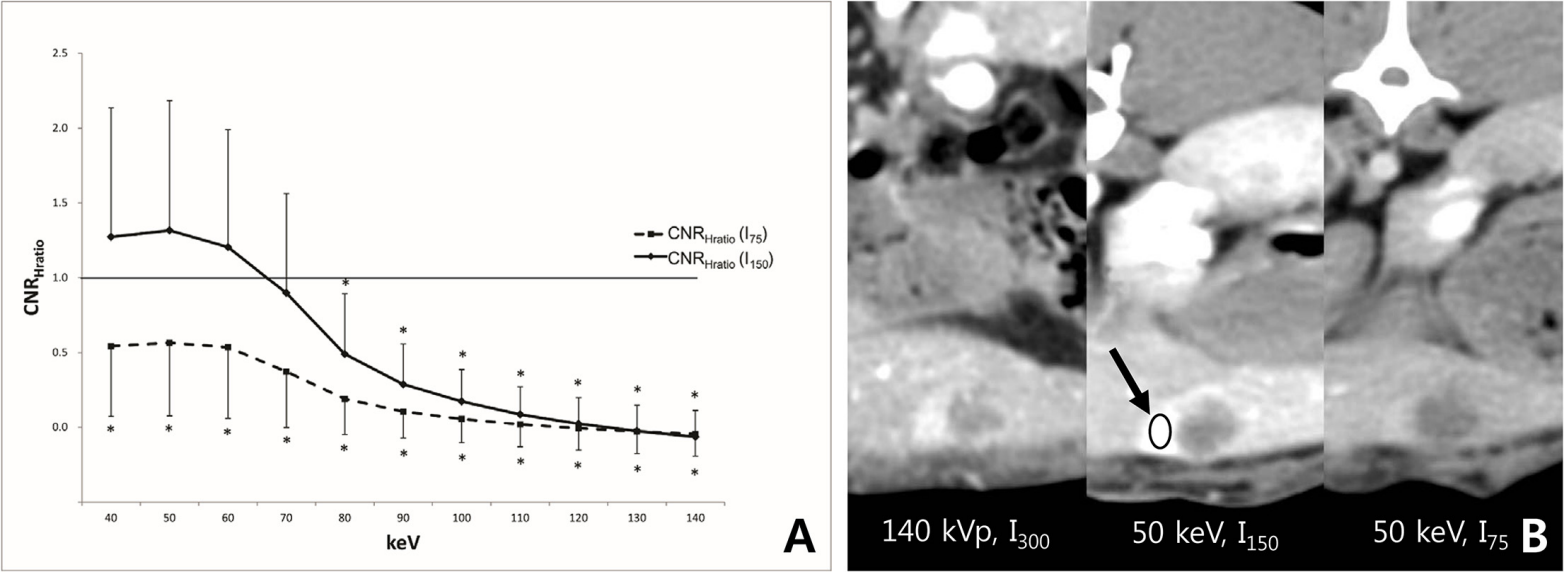
(a) The mean CNR_Hratio_ with I_150_ was not different from 1 between 40keV and 70 keV, whereas, the mean CNR_ratio_ with I_75_ was significantly lower than 1 for the entire keV range of monoenergetic images. (b) Compared to the CNR_H_ of 140 kVp with I_300_, the CNR_H_ was higher in the 50 keV image with I_150_ (CNR_Hratio_ = 1.2), whereas the CHR_H_ was lower in the 50 keV image with I_75_ (CNR_Hratio_ = 0.2). Note.—*, CNR_Hratio_ was significantly lower than 1; arrow, and the region of interest was drawn in the hypervascular portion of the VX2 tumor.

For hypervascular lesions, the CNR_Hratio_ with I_150_ was significantly lower than 1 from 80 keV to 140 keV, whereas there was no significant difference from 40 keV to 70 keV (P>0.999, respectively). The CNR_Hratio_ with I_75_ was significantly lower than 1 for the entire keV range of the monoenergetic image sets (Fig 4 and Table 2). These results suggest that CM dose can be reduced to half of the standard-of-care CM dose while preserving the CNR for hypervascular FLLs in monoenergetic images sets between 40 keV and 70 keV. However, when CM dose is reduced to one-fourth of the standard-of-care CM dose, the CNR of hypervascular FLL cannot be preserved in any energy level of the monoenergetic image sets.

**Table 2:** CNR_ratio_ in monoenergetic images.

keV	CNR_Hratio (I150)_	*P* value	CNR_Hratio (I75)_	*P* value	CNR_Lratio (I150)_	*P* value	CNR_Lratio (I75)_	*P* value
40	1.3 ± 0.9	>0.999	0.5 ± 0.5	0.001	1.1 ± 0.4	>0.999	0.9 ± 0.4	>0.999
50	1.3 ± 0.9	>0.999	0.6 ± 0.5	0.003	1.3 ± 0.5	0.062	1.0 ± 0.5	>0.999
60	1.2 ± 0.8	>0.999	0.5 ± 0.5	0.001	1.4 ± 0.5	0.005	1.1 ± 0.6	>0.999
70	0.9 ± 0.7	>0.999	0.4 ± 0.4	<0.001	1.3 ± 0.5	0.017	1.0 ± 0.5	>0.999
80	0.5 ± 0.4	<0.001	0.2 ± 0.2	<0.001	0.9 ± 0.3	>0.999	0.7 ±0.3	<0.001
90	0.3 ± 0.3	<0.001	0.1 ± 0.2	<0.001	0.8 ±0.3	0.003	0.5 ± 0.2	<0.001
100	0.2 ± 0.2	<0.001	0.1 ± 0.2	<0.001	0.7 ± 0.3	<0.001	0.5 ± 0.2	<0.001
110	0.1 ± 0.2	<0.001	0.0 ± 0.1	<0.001	0.6 ± 0.2	<0.001	0.4 ± 0.2	<0.001
120	0.0 ± 0.2	<0.001	0.0 ± 0.1	<0.001	0.6 ± 0.2	<0.001	0.4 ± 0.2	<0.001
130	0.0 ± 0.2	<0.001	0.0 ± 0.1	<0.001	0.6 ± 0.2	<0.001	0.4 ± 0.2	<0.001
140	-0.1 ± 0.2	<0.001	0.0 ± 0.1	<0.001	0.6 ± 0.2	<0.001	0.4 ± 0.2	<0.001

**Fig 5:**
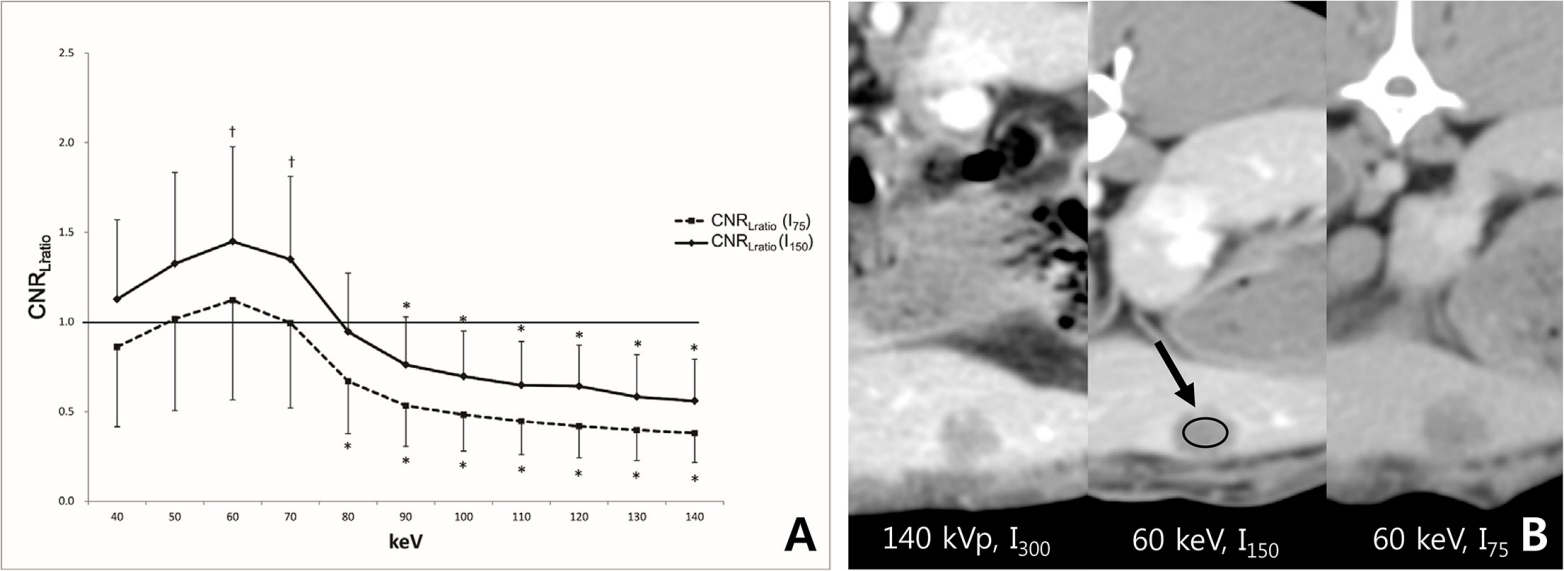
(a) The mean CNR_Lratio_ with I_150_ was significantly higher or no different to 1 between 40 keV and 80 keV. There was no significant difference in the mean CNR_Lratio_ with I_75_ from 40keV to 70keV, whereas it was significantly lower than 1 from 80 keV and 140 keV. (b) Compared to the CNR_L_ of 140 kVp with I_300_, the CNR_L_ was higher in both the 60 keV image with I_150_ (CNR_Hratio_ = 1.9) and the 60 keV image with I_75_ (CNR_Hratio_ = 1.4). Note.—*, CNR_Lratio_ was significantly lower than 1; †, CNR_Lratio_ was significantly higher than 1; arrow, the region of interest was drawn in the hypovascular portion of the VX2 tumor

In terms of hypovascular lesions, the CNR_Lratio_ with I_150_ was significantly higher than 1 between 50 keV and 70 keV and had no significant difference at 40 keV and 80 keV. There was no significant difference in the CNR_Lratio_ with I_75_ from 40keV to 70keV, whereas the CNR_Lratio_ was significantly lower than 1 from 80 keV to 140 keV (Fig 5 and Table 2). Hence, CM dose can be reduced to one- fourth of the standard-of-care CM dose for the evaluation of hypovascular FLL with 40–70keV monoenergetic image sets.

## Discussion

Our results showed that the concentration of iodinated CM could be halved in the diagnosis of hypervascular lesions and that it could even be reduced to one-fourth of its original amount for hypovascular lesions by using monoenergetic images between 40 keV and 70 keV, with preservation of the CNR. This result could be applied in patients with borderline renal function or renal failure to obtain diagnostically appropriate contrast enhanced CT images with decreased iodinated CM.

Applying the knowledge that iodine is more attenuated in lower kVp images, a lower kVp was first used to increase lesion conspicuity on CT or to decrease radiation dose while preserving the CNR of hypervascular FLLs [16, 17, 26]. Marin et al. [17] reported that a low tube voltage—high tube current protocol could improve CNR and the conspicuity of hypervascular FLLs on the late arterial phase. In this study, the CNR of hypervascular FLLs was increased 4.8 folds or if the CNR remained constant, the effective radiation dose was reduced 5.5 folds in 80 kVp with 675 mA protocols, compared to 140 kVp with 385 mA protocols. In another study, the HU of hypervascular FLL and the sensitivity for detecting hypervascular FLL was significantly higher in low kVp images of dual-source dual-energy CT than that in high kVp images [16]. Increased HU of iodine in low kVp can also be used to decrease the amount of CM with preservation of the CNR. Takahashi et al. [27] reported that the TLC of HCC in the arterial phase was not significantly different between 120 kVp with 600 mg I/mL and 100 kVp with 480 mg I/mL images for all sizes and the TLC was even better for HCC smaller than 1 cm in 100 kVp with 480mg I/mL images. In a study by Nakayama et al. [18], HU of the aorta, liver, pancreas, spleen, renal cortex and gallbladder was significantly higher in 90 kVp than in 120 kVp in the early arterial phase even though iodinated CM dose was reduced by 20% and in some cases, the effective dose was reduced by 50% at most. Most of these studies adapted polychromatic CT images, either obtained from dual-energy CT or single-energy CT and this method could reduce not only CM dose, but also radiation exposure by decreasing tube voltage. However, polychromatic images can be limited because image noise inevitably increases as tube voltage decreases due to the reduced photon flux [16–18, 26, 28]. According to a previous report, image noise was almost doubled when tube voltage was decreased from 140 kVp to 80 kVp [17]. Hence, the reduction of CM dose was limited only up to 20% [17, 27]. In this study, we tried to focus on reducing the dose of iodinated CM as much as possible while preserving image quality for patients with impaired renal function. We adopted monoenergetic images and maximized the HU increase of iodine CM with tube voltage reduction. This reduced CM doses up to 50% for hypervascular lesions while preserving the CNR of FLLs.

Lower tube voltage images also have advantages in the evaluation of hypovascular lesions because the HU of liver parenchyma is relatively more increased in lower tube voltage images compared to that of hypovascular lesions [18, 19]. Robinson et al. [19] reported that images obtained with 80 kVp showed more HU difference and better lesion conspicuity compared to those obtained with 120 kVp for the evaluation of hypovascular liver metastasis. In a study by Yamada et al, the CNR of hypovascular metastasis had its highest value in 68 keV monoenergetic images [20]. This result was comparable with ours in that the CNR was the highest in 60–70 keV monoenergetic images with I_150_. Furthermore, in contrast to hypervascular FLLs, the CM dose can be reduced to one-fourth of its original amount while preserving the CNR by using 40–70 keV monoenergetic images for the evaluation of hypovascular FLLs.

Radiation dose increased in dual-energy CT from 20% up to a maximum of 100% depending on scanning parameters and the field of view [21, 29]. Although radiation dose reduction has recently become an important issue of interest, decreasing the amount of iodinated CM might be more clinically important than reducing radiation dose for patients who have significant risk factors for CIN or who have decreased renal function. If contrast enhancement is essential for the accurate diagnosis of patients with impaired renal function, physicians or radiologists should evaluate the individual risks and benefits, and if clinically indicated, contrast enhanced CT can be performed with minimum iodinated CM by using GSI dual-energy CT despite the possible increase of radiation exposure. Applying iterative reconstruction to dual-energy CT might be another way to reduce radiation dose [27, 30, 31]. However, it does not allow the separate adjustment of CT parameters and only preset protocols can be chosen in GSI dual-energy CT, which is why the low dose CT protocol was of limited use until now. Furthermore, automatic exposure control is not yet available in GSI dual-energy CT. Dose reduction techniques should be applied in GSI dual-energy CT in the future for a more wide application of contrast enhanced CT with low dose CM.

There are several limitations in our study. First, quantitative analysis with variables such as the CNR does not always represent diagnostic performance. Furthermore, because animal models with only one or two lesions in the liver were used, diagnostic sensitivity for detecting FLL could not be evaluated compared to pathological gold standard. Hence, prospective clinical studies in humans that evaluate diagnostic performance are warranted. Second, body habitus and hemodynamics may be considerably different between humans and rabbits. Third, our results might be affected by the injection protocol of the CM, although the fixed injection duration method has been adopted recently because of its accurate prediction of the optimal window of the LAP and HVP

In conclusion, with GSI dual-energy CT, the concentration of iodinated CM (or the total amount of iodine CM) might be halved in the diagnosis of hypervascular liver lesions and reduced to one-fourth in the diagnosis of hypovascular liver lesions, while preserving CNRs of FLLs. Although this study assessed the feasibility of GSI dual-energy CT for reducing CM dose with animals, the results might be the basis for another CIN prevention method other than volume expansion or medication.
